# An Unusual Therapy for an Atypical Case of Secondary Extranodal, Non-parenchymal Central Nervous System Marginal Zone Lymphoma

**DOI:** 10.7759/cureus.79468

**Published:** 2025-02-22

**Authors:** Siham Ahchouch, Abdelilah El Barrichi, Mohammed Allaoui, Hicham El Maaroufi, Kamal Doghmi

**Affiliations:** 1 Clinical Hematology, Avicenne Military Hospital, Cadi Ayyad University, Marrakech, MAR; 2 Clinical Hematology, Mohammed V Military Teaching Hospital, Mohammed V University, Rabat, MAR; 3 Pathology, Mohammed V Military Teaching Hospital, Rabat, MAR; 4 Hematology, Mohammed V Military Hospital, Rabat, MAR

**Keywords:** b-cell marginal zone lymphoma, cavernous sinus (cs), exophthalmos, high dose methotrexate regimen, mr-chop regimen, scnsl, secondary central nervous system lymphoma, secondary cns mzl

## Abstract

Marginal zone lymphomas (MZL) are low-grade B-cell neoplasms with indolent clinical behavior and a favorable prognosis. Central nervous system (CNS) involvement is extremely rare in MZL, as in other low-grade lymphomas where some presented with primary CNS disease without involvement elsewhere, and only a few cases were secondary to MZL. The dura mater is the most common site of involvement, while cavernous sinus involvement is extremely rare. There are no specific treatment recommendations for patients with secondary CNS-MZL exhibiting an indolent course. Therapeutic trials and recommendations typically focus on aggressive lymphomas with a poor prognosis, where induction with intensive chemotherapy, including high-dose methotrexate and/or cytarabine, followed by autologous stem cell transplantation, is standard. We present the case of a 50-year-old man with MZL involving the cavernous sinus, who initially presented with neurological and ophthalmological symptoms and was successfully treated with high-dose methotrexate and the CHOP (cyclophosphamide, doxorubicin, vincristine, and prednisone) regimen.

## Introduction

Central nervous system lymphoma (CNSL) is rare, affecting less than 5% of patients [1], and includes a variety of subtypes, each with different clinical findings, treatment approaches, and prognoses. Among these, marginal zone lymphoma (MZL) is the second most common after diffuse large B-cell lymphoma (DLBCL) [2]. MZL is a group of mature, indolent B-cell lymphomas that account for up to 15% of non-Hodgkin lymphomas (NHL) [2]. It arises from memory B cells in the "marginal" zones of secondary lymphoid tissues and is characterized by indolent clinical behavior and a favorable prognosis. The fifth edition of the World Health Organization (WHO) classification of lymphoid and hematopoietic tumors categorizes MZL into five subtypes based on the tissue microenvironment: extranodal MZL (EMZL) of mucosa-associated lymphoid tissue (MALT), splenic marginal zone lymphoma (SMZL), nodal marginal zone lymphoma (NMZL), pediatric marginal zone lymphoma, and primary cutaneous MZL. Depending on the location, EMZL can be further divided into gastric and nongastric MALT lymphoma. The latter commonly occurs in ocular appendages, skin, thyroid, lungs, salivary glands, and breasts, and rarely involves the central nervous system (CNS), either primarily or secondarily [3,4].

NHL involvement of the CNS occurs in two forms: primary CNS lymphoma (PCNSL), which accounts for 4% of all brain tumors [4], and is restricted to the brain parenchyma, vitreoretinal space, cranial nerves, leptomeninges, and, rarely, the spinal cord alone [2-5]; and secondary CNS lymphoma (SCNSL), characterized by concurrent systemic and CNS lymphoma localization. The latter may present de novo, synchronously with systemic involvement, or during relapse (isolated CNS relapse of previously treated systemic lymphoma, or relapse with concomitant systemic and CNS involvement after systemic lymphoma) [6]. We report a case of secondary CNS MALT lymphoma originating from the cavernous sinus, treated with chemotherapy alone.

## Case presentation

A 50-year-old soldier with a history of arterial hypertension and smoking (23 pack-years) was weaned off smoking six months prior to his first consultation. He also had recent use of corticosteroids for several days in the context of allergic rhinitis. He had no history of organ transplantation, blood transfusions, or immunosuppressive drug use. The patient presented with a 12-month history of progressive, spontaneous, and constant exophthalmos with horizontal diplopia, without associated blurred vision or ptosis. The diplopia was not exacerbated by physical exertion, was maximal with lateralized gaze in both directions, and improved with eye occlusion. The patient did not report retroorbital pain, dizziness, headache, or nausea. There were no signs of convulsions, paraparesis, memory disorders, or neurovegetative symptoms. He also mentioned the appearance of left-sided, centimeter-sized spinal adenopathies for over three months, with no other neurological symptoms or general signs.

An initial examination revealed an orbital syndrome with exophthalmos (not measured with the Hertel exophthalmometer) and conjunctival hyperemia. Examination of the cranial nerves showed third cranial nerve (CN) palsy, which caused horizontal binocular diplopia at a distance and limited abduction. The pupillary light reflex was intact, and there was no nystagmus. The patient did not have fever, meningeal signs, weight loss, or night sweats. There was no rash in the trigeminal territory ipsilateral to the right oculomotor nerve damage, nor were there any deficits in the trigeminal nerve distribution, sensory or motor deficits in the hemicorporeal region, cerebellar signs, or signs of generalized myasthenia gravis. There was no splenomegaly, and examination of the lymph nodes revealed left spinal lymphadenopathy, with the largest node measuring 15 mm, without any inflammatory signs. The rest of the physical examination findings were unremarkable.

**Figure 1:**
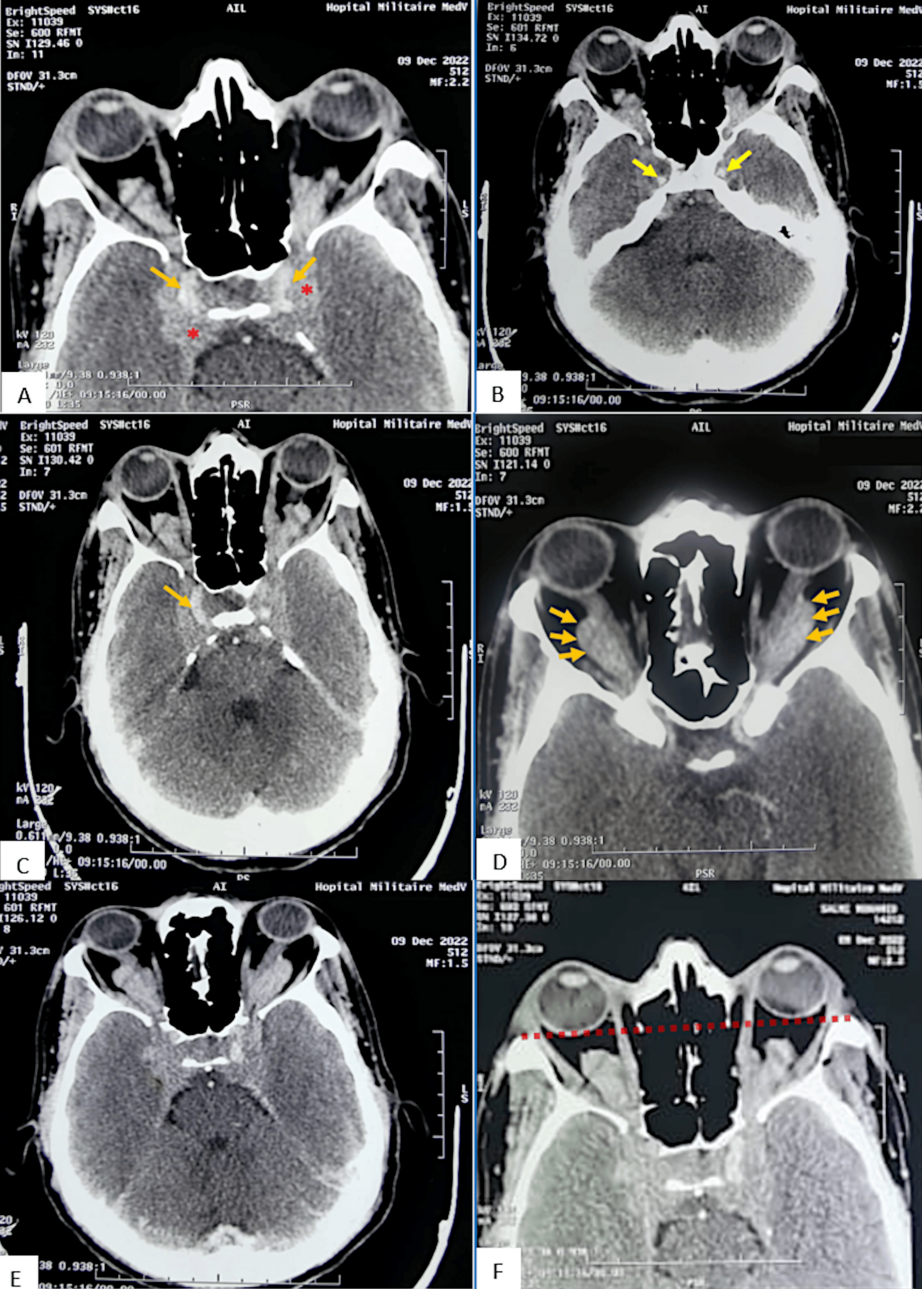
Axial CE brain CT. The CT scan revealed a 47x25 mm moderately enhancing lesional mass centered on the spheno-cavernous region (red asterisk, A, E), heterogeneous in appearance, with the invasion of the sheaths of both optic nerves (orange arrowhead, A, C, D), the superior orbital fissures, peri-nerve sheaths, and laterally into the cavernous sinuses, encompassing both carotid arteries in the intra- and supracavernous portions (yellow arrowhead, B). Grade III bilateral exophthalmos is indicated by the dotted line (F). CE, contrast‐enhanced; CT, computed tomography

**Figure 2:**
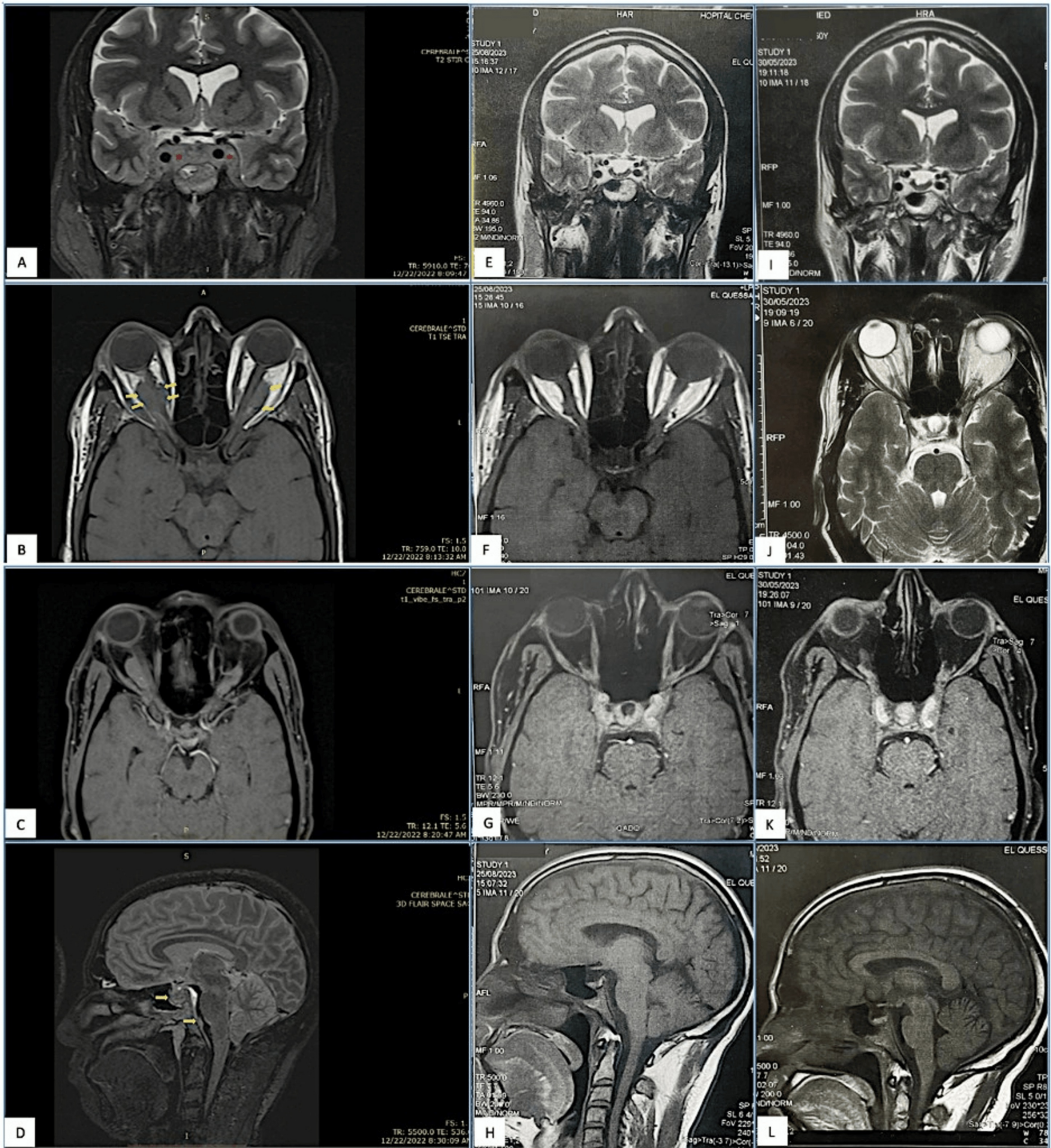
Orbital-cerebral brain MRI. AT initial evaluation (A-D): Infiltrating tumor process in the sellar region, extending into the cavernous sinuses (A). This process invades both orbital pyramids through the superior orbital fissure and encompasses the intracranial and posterior intraorbital optic nerves (yellow arrowhead B). In the sellar region, the pituitary parenchyma is surrounded on both sides by this process (C), which partially fills the optochiasmatic cistern, extending posteriorly toward the prepontine cistern (yellow arrowhead D). Screening after four cycles of chemotherapy (E-H) and after six cycles of chemotherapy (I-L). MRI, magnetic resonance imaging

Brain CT revealed a heterogeneous 47x25 mm moderately enhancing mass centered on the spheno-cavernous region, with invasion of the sheaths of both optic nerves, the superior orbital fissures, peri-nerve sheaths, and laterally into the cavernous sinuses, encompassing both carotid arteries in the supra- and intracavernous portions (Figure 1). Grade III bilateral exophthalmos was also present (Figure 1). Initial diagnoses made by the reporting neuroradiologist included meningioma or PCNSL. Orbital-cerebral magnetic resonance imaging (MRI) showed an infiltrating tumor process in the sellar region that extends into the cavernous sinuses. This process invades both orbital pyramids through the superior orbital fissure and encompasses the intracranial and posterior intraorbital optic nerves. In the sellar region, the pituitary parenchyma is surrounded bilaterally by this process, which partially fills the optochiasmatic cistern and extends posteriorly toward the prepontine cistern (Figure 2).

**Figure 3:**
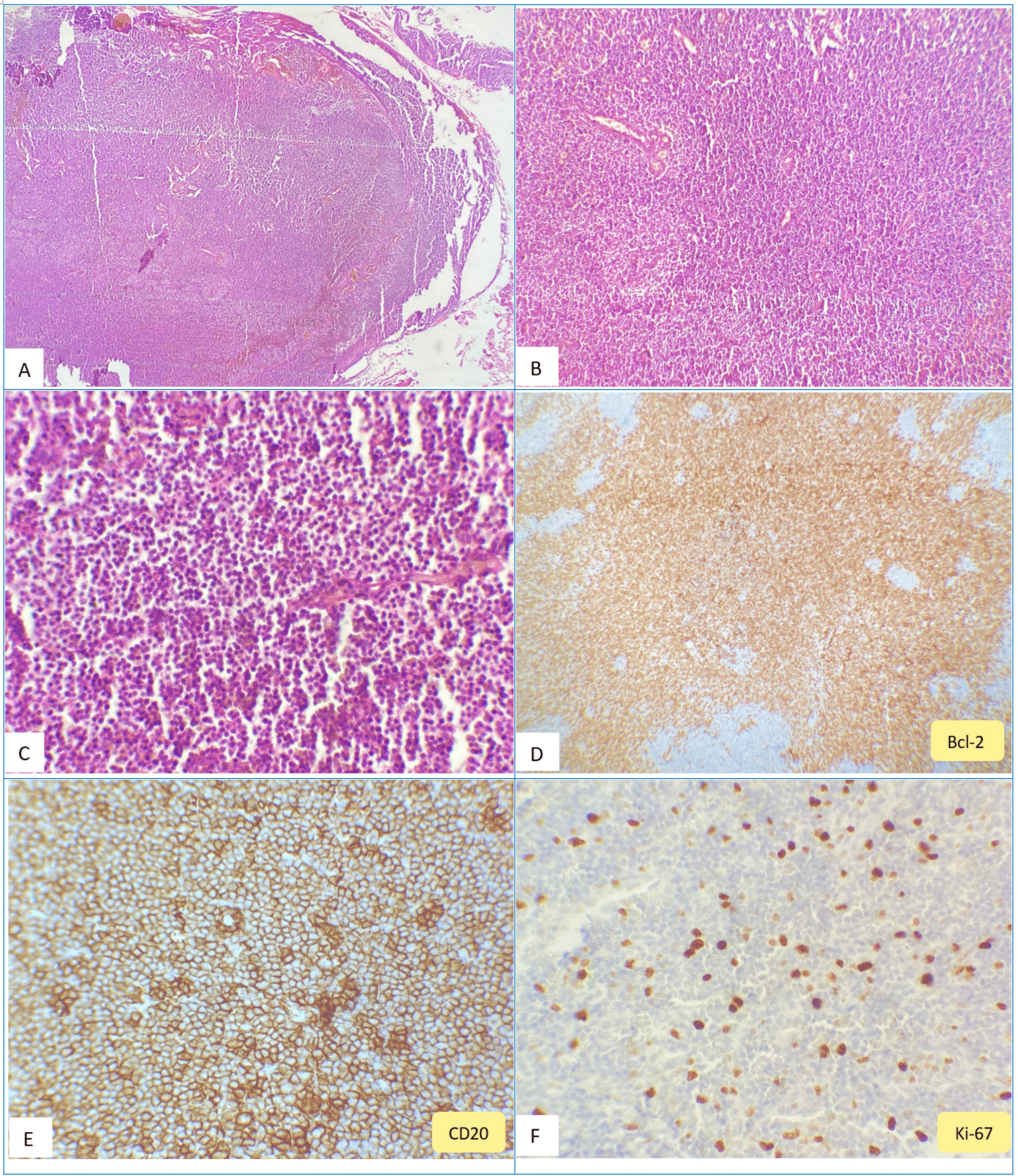
Histological and immunohistochemical features of spinal lymph node resection. (A) Lymph node parenchyma with completely obliterated architecture due to diffuse nodular lymphomatous proliferation that infiltrates adjacent fibroadipose tissue. Hematoxylin and eosin (H&E) stain, original magnification ×100. (B) These cells are arranged in diffuse patches; mitoses of small cells are rare, and no granulomas were observed. H&E, original magnification ×200. (C) Cytological details of lymphoma cells showing diffuse infiltration of small to medium-sized lymphocytes with rounded nuclei, fine chromatin, and reduced or occasionally clarified cytoplasm, mixed with plasma cells. H&E, original magnification ×400. (D, E) Immunohistochemical examination (original magnification ×400) showing positive staining for Bcl2 and CD20 (D, E) and Bcl2 (F). Ki-67 proliferation index is estimated at 25%.

An accessible spinal adenopathy biopsy was performed to avoid a more invasive procedure. Histological examination of the spinal adenopathy revealed a completely obliterated lymph node architecture, characterized by diffuse lymphomatous proliferation organized into large patches that infiltrate adjacent fibroadipose tissue. The neoplastic infiltrate consists of marginal zone B cells (monocytoid) with abundant pale cytoplasm (Figure 3). Immunohistochemical analysis showed positive staining for CD20, CD79a, Bcl2, and Bcl6, and negative staining for CD3, CD5, CD10, CD23, Cyclin D1, and SOX11. The Ki-67 proliferation index was estimated at 25%. Morphological and immunohistochemical findings were consistent with marginal zone B-cell lymphoma (Figure 3).

**Figure 4:**
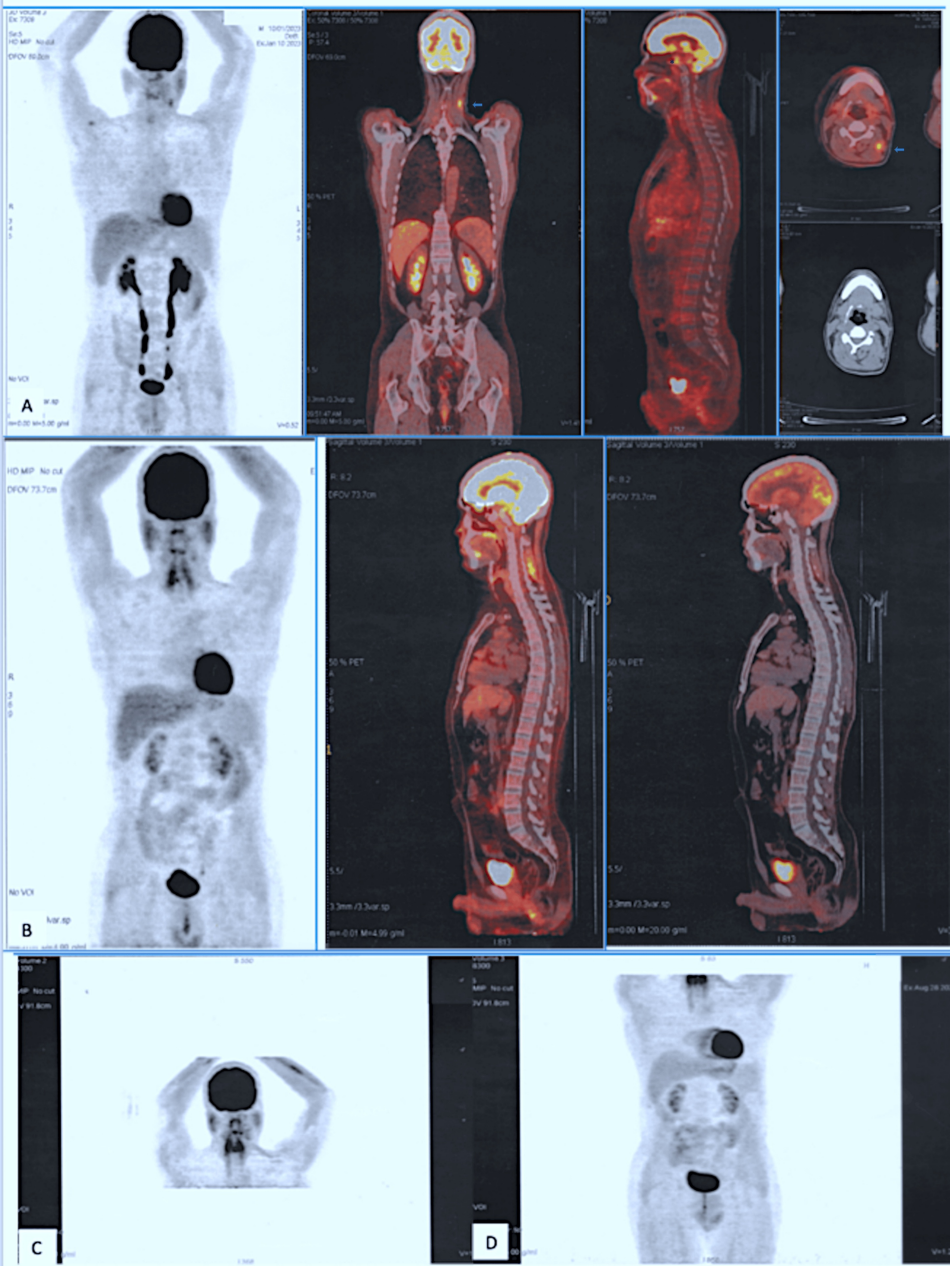
TEP-scanner images at initial evaluation (A), after two cycles of chemotherapy (B), four cycles of chemotherapy (C), and six cycles of chemotherapy (D). A: symmetrical, retro-ocular orbital hypermetabolism with hypermetabolic pathological spinal ganglia (blue arrows). PET, positron emission tomography

A complimentary positron emission tomography (PET) scan performed one month after diagnosis revealed bilateral, almost symmetrical, retro-ocular orbital hypermetabolism with small suspicious hypermetabolic pathological foci in the left spinal ganglia (Figure 4). During the initial evaluation of the patient, no total-body CT and bone marrow biopsy were performed. The patient has undergone routine laboratory tests, including a complete blood count and cerebrospinal fluid (CSF) examination (Table 1). In view of this very slowly evolving picture, the diagnosis of primary cerebral MZL was made with the onset of the locoregional invasion stage IIE. Given the recent involvement of peripheral spinal lymph nodes adjacent to cerebral involvement and the recent use of low-dose corticosteroids, systematic chemotherapy was decided, including high-dose methotrexate instead of localized radiation therapy. The patient received six cycles of MR-CHOP (methotrexate, rituximab, cyclophosphamide, doxorubicin, vincristine, and prednisone) (21-day cycle) with partial remission on intermediate MRI after four cycles (Figures 2). Given the disappearance of clinical symptoms, the patient's young age, and the excellent response to treatment, the last two courses were administered without anthracyclines, using the MR-RCVP (methotrexate, rituximab, cyclophosphamide, vincristine, and prednisone) regimen. The patient is in complete metabolic remission (Figures 3), and the disease remains stable on brain MRI (Figure 2). We recommend surveillance rather than adjunctive radiation therapy. Currently, seven months after the end of treatment, the patient remains asymptomatic.

**Table 1:** Routine laboratory test results. *Capillary glycemia concomitant to lumbar puncture ^$^Using MDRDs formula Ag: antigen; ALT: alalanine deshydrogease; AST: aspartate transaminase; CSF: cerebrospinal fluid; HBV: hepatitis B virus; HCV: hepatitis C virus; HIV: human immunodeficiency virus; LDH: lactate dehydrogenase; RBC: red blood cell; WCCL: white cell count level

Blood test	Results	Normal range
CSF cells analysis		
Appearance	Clear rock water	
Cytology	No malignant cells	
WCCL, E/mm^3^	4	5-10
RBC, E/mm^3^	3	0-10
Biochemical study		
Protein level, g/L	0.54	0.15-0.45
CSF glucose	0.74	0.40-0.70
Serum glucose,^* ^g/L	0.85	0.7-1.10
Flow cytometry	22% lymphocytes B 19+/CD10- CD20^Low^	
	75% lymphocytes T CD3+ (55% CD4+ 40% CD8+)	
Complete blood count: white blood cell count, x10^3^/µL	7.3	4-10
Neutrophil, x10^3^/µL	4.4	1.5-7.5
Lymphocytes, x10^3^/µL	2.1	1.5-4.0
Hemoglobin level, g/dL	16.6	12-16
Platelet count, x10^3^/µL	257	150-450
Vitamin B12, pg/mL	321	187-883
Chemiluminescence (Alinity ci)		
Serum folate, ng/mL	4.00	3.10-20.00
Chemiluminescence (Alinity ci)		
C-reactive protein, mg/L	<1.00	<5.0
Turbidumetry (Alinity ci)		
Serum protein electrophoresis	Normal qualitative and quantitative electrophoretic profile	
Albumine, g/L	58.1	60-80
Gammaglobulins, g/L	14.14	8-13
Capillary electrophoresis (capillaries)		
LDH, U/L	159	125-243
Turbidumetry (Alinity ci)		
B_2 _microglobulin, mg/L; turbidimetry (Alinity ci)	1.93	0.97-2.64
Serology		
VHB		
Ac anti-HBs, mUI/mL	Negative (3.5)	Index E/VS<1.00
HVB Ag HBs	Negative Ratio (S/CO 0.21)	
Ac anti-HBc totaux	Negative Index (E/VS 0.07)	Index E/VS<1.00
VHC Ac anti-HVC	Negative	<1.00
HIV (Ac anti-VIH1+2, AgP24)	S/CO 0.21	Index E/VS<1.00
Chemiluminescence (Architect i2000, Abbott)		
AST, U/L	13	0-35
ALT, UI/L	16	0-40
Creatinine, mg/L	8	6-13
Creatinine clearance, mL/min^$^	102	90-130

## Discussion

SCNSL is characterized by involvement of the CNS due to NHL, either at the time of initial diagnosis or during relapse (relapse of lymphoma in the CNS, with or without concurrent involvement of systemic lymphoma) [7].

Secondary involvement of the CNS occurs in 2-5% of cases among patients with diffuse large B-cell lymphoma [8] and in 5% to 40% of cases of Burkitt lymphoma (BL), which is associated with a high risk of CNS involvement [8-10]. Invasion by mantle cell lymphoma, peripheral T-cell lymphoma, and anaplastic large cell lymphoma has also been described [11]. However, CNS involvement is rarely observed in indolent lymphomas, such as follicular lymphoma [5] and chronic lymphocytic leukemia [10,12,13]. In a recent SEER analysis, the age-adjusted incidence rate for patients with CNS involvement was approximately 0.03 per 1,000,000 person-years [14]. Primary and secondary CNS involvement by low-grade lymphomas, such as MZL, is rare. Approximately 207 cases of primary CNS MZL (PCNSMZL) have been reported [15], and only 32 cases of secondary CNS MZL (SCNSMZL) have been found in the literature (Table 1) [16], including our case and a case of MZL with signs of transformation to aggressive lymphoma, treated as such [17].

MZL is considered an indolent lymphoma [18] that generally occurs in association with autoimmune diseases and chronic inflammation, with the most common site being the gastrointestinal tract due to autoantigenic stimulation, particularly from Helicobacter pylori. The cells of origin can also be the mucosa of nongastrointestinal organs such as the lungs, bladder, salivary glands, conjunctiva, and lacrimal glands, or more rarely, nonmucosal tissue sites such as the liver, breast, thyroid, orbit, skin, and CNS. In this case, CNS MZL can originate in the meningoepithelial cells of the arachnoid villi of the dural venous sinuses, where MALT-like tissue arises due to chronic inflammation and the local accumulation and proliferation of antigen-dependent B cells, leading to the development of lymphoma [19,20].

In CNS MZL, a female preponderance has been described, though this trend may be less pronounced in secondary MZL, where the female-to-male ratio is lower [13]. Regarding age distribution, although the average age at diagnosis for all CNS MZL cases in published studies is 60 years [13,21,22], secondary CNS MZL appears to affect a slightly older population. In fact, the median age at the time of primary disease diagnosis is 51 years, while for secondary disease, it is 62 years [13].

The mode of onset of SCNSMZL is similar to that of all indolent lymphomas with neurological involvement, characterized by insidious, chronic neurological symptoms that typically last several months. This is due, on one hand, to cerebral plasticity and, on the other, to the slow growth of the intracerebral process, which progressively leads to a mass effect.

In fact, as in SCNSMZL, the predominant initial symptoms that prompt consultation depend on the location of the tumor, such as visual disturbances due to compression of the optic nerves, invasion of the eyeballs, and exophthalmos, with or without oculomotor disturbances in nearly a third of cases, as seen in our patient [13,19,23,24]. Other reported symptoms include headache, CN damage, neurovegetative disorders [13,19,25,26], limb dysesthesia, muscle weakness, seizures [13,27-30], episodes of confusion, psychiatric disorders, personality changes, and cognitive and memory problems [13,25,30]. In most cases, CNS symptoms were the trigger for the diagnosis of systemic disease, and only two cases of SCNSMZL have been described with general signs of systemic disease and tumor syndrome at the time of diagnosis [13,31].

The most common locations for cerebral lymphoma are the dural, periventricular, or deep parenchymal regions of the brain [13]. PCNSMZL typically presents as extra-axial masses implanted in the dura or, less commonly, as intraparenchymal lesions that develop in perivascular locations [15]. In the largest series of 26 patients recently published by Sunderland et al., patients with primary CNS MZL typically present as a single mass arising from the dura without involvement of systemic disease. In contrast, SCNSMZL presents with more varied and heterogeneous locations, suggesting that SCNSMZL is more likely to involve the parenchyma and leptomeninges [19,32].

SCNSMZL infiltrates, in order of frequency, the dura mater in 13 cases, primarily in the frontal region and at the level of the optic nerves [13,19,24,26,29-31], and parenchymal involvement with different localizations (frontal, frontoparietal, temporal, parieto-occipital, cerebellar, cervical, and thoracic spinal cord) [13,23,27,28]. Two cases of periventricular involvement [13,25] and two other cases involving concomitant retinal and choroidal involvement, as well as vitreous and choroidal involvement, have also been reported in the literature [19]. In Angelpoulou's series, a patient with visual disturbances and bilateral papilledema was diagnosed with SCNSMZL, with CSF being the only positive diagnostic finding [23]. A single case of left optic nerve involvement was reported in the Sunderland et al. series, without further specification of the extent of intracranial invasion [13]. In our case, the process infiltrated the sellar region, including the cavernous sinus, without associated dural involvement or invasion of the optic nerve with cavernous sinus involvement (Table 2).

**Table 2:** Patient characteristics, treatment, and outcome. Allo-HSCT: allogeneic hematopoietic stem cell transplantation; Ara-C: cytarabine; biw: twice weekly; BM: bone marrow; CNS: central nervous system; COP: cyclophosphamide, vincristine, prednisolone; CR: complete response; CRu: complete response unconfirmed; CS: corticosteroids; CSF: cerebrospinal fluid; CT: computed tomography; CTx, chemotherapy; CVPR: cyclophosphamide, vincristine, prednisolone, rituximab; Dexa: dexamethasone; Doxo: doxorubicin; F: female; i.t.: Intrathecal; i.v.: intravenous; IFO: ifosfamide; IPT: immunophenotyping; i.t.: intrathecal; LN: lymph node; M: male; MBL: monoclonal B lymphocytosis; MRI: magnetic resonance imaging; MTX: methotrexate; MZL: marginal zone lymphoma; NA: not available; OS: overall survival; PET: positron emission tomography; PR: partial response; R-CHOP: rituximab, cyclophosphamide, vincristine, doxorubicin, prednisolone; R-COP: rituximab, cyclophosphamide, vincristine, prednisolone; R: rituximab; RTx: radiotherapy; Sx: surgery; VCR: vincristine

Ref.	Year	No Case	Age/sex	Presentation mode	Clinical features	Oculocerebral location and imaging	CSF/biopsy of CNS mass	Systemic location/pathology of the systemic lesion	Treatment	CNS response, outcome, OS (y)
[7]	2020	Case 1	77/M	Simultaneously	Visual loss	Left optic nerve	NA	NA	CTx (RCHOP+ MTX (i.t.))	NA, died, 0.1
		Case 2	59/M	Simultaneously	Scalp swelling, dysarthria	Right frontoparietal dura	NA	NA	CTx (CVPR)	CR, alive, 11.44
		Case 3	66/F	Simultaneously	Headaches, fevers, and fatigue	Frontal lobe, occipital lobe, and cerebellum	NA	NA	CTx (MTX(i.t.)+Arac (i.t.))	PR, died, 0.583
		Case 4	53/F	Simultaneously	Unknown	Left frontal lobe	NA	NA	RTx	PR, died, 1.74
		Case 5	66/M	Simultaneously	Visual changes	Right posterior parieto- occipital lobe	NA	NA	Sx+RTx	NA, died, 0.16
		Case 6	67/F	Simultaneously	Unknown	Unknown	NA	NA	Unknown	Unknown, alive, 3.01
		Case 7	67/M	Simultaneously	Unknown	Unknown	NA	NA	Unknown	PR, alive, 1.34
		Case 8	46/F	Simultaneously	Unknown	Unknown	NA	NA	CTx (FDR+Ara-C)	CRu, alive, 3.45
		Case 9	57/F	Simultaneously	Personality change, unsteadiness, memory loss	Surrounding lateral and third ventricles	NA	NA	CTx (IFO+Ara-C+MTX)	Unknown, alive, unknown
		Case 10	34/M	Simultaneously	Altered sensation	Dural-based sacral spinal canal	NA	NA	Sx, CTx (RCHOP), RTx	CR, alive,4.22
		Case 11	62/M	Simultaneously	Headaches, pain in the V2 distribution of the trigeminal nerve	Dura overlying optic nerves, pterygopalatine fossa, CS, optic chiasm	NA	NA	Sx, CTx (R)	Progressive, alive, 1.57
		Case 12	69/F	Simultaneously	Visual changes	Dura of right orbit and skull base	NA	NA	Sx, RTx	Progressive, alive, 1.6
		Case 13	60/F	Simultaneously	Altered sensation	Skull base leptomeninges and extradural space C2-C4	NA	NA	Sx, CTx (R+Ibrutinib)	Unknown, alive, 0.09
[8]	2020	Case 1	80/M	Simultaneously	Confusion, irritability, word-finding difficulties, impaired concentration, disruption of circadian rhythm, mild action tremor of the upper limbs, subtle postural instability	MRI-brain: periventricular + subcortical white matter lesions	CSF: Monoclonal B-cell population identical to a known marginal zone clone	PB: MBL: indolent clone of marginal zone origin (80%) and a smaller CLL-like phenotype clone (7%); CT-scan: mild splenomegaly	CTx (Oral dexa+4 MTX (i.t.)+6R-COP)	PR, alive, 2
[9]	2019	Case 1	51/M	5 year, CNS relapse of systemic MZL	Visual disturbances with bilateral papilledema	MRI brain: normal	CSF: Monoclonal small lymphocyte population CD20+, FMC7+, CD79a+, CD5-, CD23+, CD25-, surface κ-light chain+, annexin-1-, and CD11c-	Blood, BM (15-20%) involvement by small lymphocytes with the same IPT	CTx (8 i.t. (Ara-C, Dexa MTX)+8 i.v. R (375 mg/m^2^)+3 i.t. R (25 mg) every 3 days)	CR, alive, 8.67
		Case 2	54/F	Simultaneously	Blurred vision with bilateral papilledema	Periventricular white matter of the left frontal lobe + multiple demyelinating lesions in the left frontal lobe, cervical and thoracic spinal cord	CSF: positive predominance of CD20+, kappa monoclonal B-lymphocytes	Blood, BM: MZL: 15% of monoclonal B-cells CD20+, kappa+, CD22+, CD19+, CD79b+, CD5-, CD10-, CD23-, FMC7+, and CD200-	CTx (6 days of HD-CS+2 i.t (MTX/dexa )+9 i.t. R (25 mg bi-w)+6 i.v. R (375 mg/m^2^))	CR, alive, 2
[10]	2014	Case 1	55/F	18 months, relapse after systemic MZL (stage IV, extranodal)	Right upper and lower limb weakness	Non-contrast cerebral CT-scan: a large, hyperdense, left temporal mass, 3.2x3.5 cm, with perilesional edema, mild midline shift, and mild compression of the left ventricle	Brain lesion biopsy: infiltrating neoplastic lymphoid cells small to medium size CD20+, CD79a+, CD43+, CD3-, CD5-, CD10-, CD21-, CD23-, cyclinD1-	Blood, BM aspiration/biopsy dehydrogenase: negative; CT scans: no recurrent lymphoma	CTx (1 cycle:HD-MTX+cyclophosphamide+Doxo i.v.+i.t.(Ara-C, Dexa/2 cycles: HD-MTX+Ara-C+Doxo+IFO+VCR+VP-16 i.v.+ i.t (Ara C, Dexa, MTX))	CR, alive, NA
[11]	2012	Case 1	42/M	Simultaneously	Paraplegia, reduced vibration sense, bilateral neuropathic pain in the legs	Temporal lobe lesion (9 mm), cauda equina meningeal involvement	CSF: MZL cells κ-λ light chain expression +	Blood, LN, spleen: SMZL (CD19+, CD20+, cy79a+, sIgM+, FMC7+, λlight chain, CD5± CD38±, CD10-, CD11c-, CD23-, CD103-, CD138-	CTx (HD Ara-C/HD-MTX i.v.+ i.t. (MTX/Ara C/Dexa)+allo-HSCT)	CR, alive, 3
[12]	2010	Case 1	NA	Simultaneously	Headache, blurred vision, facial numbness, hearing loss, 5 months	Optic nerve involvement with extension to dura mater; MRI brain: abnormal dural thickening + enhancement at the level of tentorium extending to cavernous sinus and right middle cranial fossa	NA	Bilateral orbital MZBCL; CD20+, CD5-/CD10-/ CD23-/cyclinD1-	RTx (IMRT (30 Gy))	CR, alive, 0.58
		Case 2	NA	4 years later	Diplopia, blurred vision, periorbital edema, proptosis and limitation of external eye movements, 4 months	Optic nerve involvement with extension to dura mater: -; MRI brain: left intraorbital mass (12×6 cm) encasing the optic nerve with extension to the extraocular muscles, left cavernous sinus and infratemporal fossa	CSF: positive	Orbital MZBCL: IgG λ chain restricted, CD20+ CD10/CD5/ cyclinD1-	CTx (DeAngelis protocol (5 cycles)+RCVP (6 cycles)+i.t. (MTX)	CR, alive, 2 years
		Case 3	NA	5 years later	Blurry vision, conjunctival injection and bleeding, 6 months	Bilateral retinal and choroidal involvement: -; MRI brain: unremarkable	NA	Bilateral intraocular MZL: IgG Ќ chain restriction, CD20+, CD5-/ CD10-/ CD23-/cyclinD1-	Sx (resection of eyelid lesion), +RTx (24 Gy). Isolated intraocular relapse: Sx (bilateral vitrectomy)+orbital RTx (30 Gy)+CTx (DeAngelis protocol (6 cycles))	CR, alive, 3
		Case 4	NA	5 years later	Blurry vision, conjunctival injection, 1 month	Bilateral choroidal and vitreous involvement: -; MRI brain: unremarkable	NA	Bilateral intraocular MZL; IgG Ќ chain restriction, CD20+, CD5/CD10/ cyclinD1-; BM: involvement + CT scan: mildly enlarged spleen (13 cm)	Splenic MZL: conservative management: Isolated intraocular relapse: Sx (bilateral vitrectomy); orbital RTx (30 Gy); CTx (DeAngelis protocol (1 cycle); thiotepa-based regimen (5 cycles))	CR in the eyes/brain; systemic relapse (BM and spleen) at 3 years
[13]	2010	Case 1	46/F	Simultaneously	Pressure symptoms in the right frontal region of the head, blurring of vision of the right eye. Nodule in the right upper palpebral location, shotty inguinal nodes	Cerebral MRI: 2x6x6 cm extra-axial enhancing mass along the right frontal convexity, extending to the superior ridge of the orbit, across the midline with thickening and enhancement of the left frontal dura mater. Increased T2 signal in the adjacent cortical sulci with subtle contrast enhancement	Dural mass resection: MALT lymphoma expressing CD20, CD52, CD19, and CD38, CD5-. SCF: negative	LN biopsy: lymphoid proliferation, κ-restricted B-cell neoplastic proliferation. Orbital mass biopsy: lacrimal gland involvement by MALT lymphoma with the same IPT and PCR as the LN and the dural mass. BM: small population (2.6%) of kappa-restricted B-lymphocytes; PET-CT scan: shotty cervical, axillary, and inguinal lymph nodes with the highest values in inguinal regions (SUVmax: 2-4 range)	CTx (IVR, fludarabine, mitoxantrone (4 cycles)); Relapse within one year: CTx (R maintenance)	CR, alive, 2.75
[14]	2006	Case 1	73/M	Simultaneously	Grade 4 weakness in her right arm and an expressive dysphasia	Cranial CT scan: homogeneously enhancing left frontoparietal mass with a wide dural base, causing mass effect	CSF: MZL: CD10-, Mib-1 10%, Bcl-1-, Bcl-2+, CD5-, CD20+, and CD79+	BM: large cell transformation. CT scan: presacral mass with similar histology	Sx (excision of the tumor)+CTx(CS) i.t (Arac+MTX)+Chlorambucil).	NA
[15]	2005	Case 1	53/M	Simultaneously	Transitory episodes of bilateral visual loss. Bilateral papilledema	Brain CT scan: triventricular hydrocephalus; cerebral MRI: tumoral infiltration in optic nerves and the cervicodorsal meninges	CSF: B-lineage markers CD19+, CD20+, CD79b+, surface Ig κ expression, IgD+, CD5, CD23-, CD10-, CD25-, CD103-, and CD11c	PB: lymphocytes with villous morphology; BM: nodular + interstitial infiltration of small lymphocytes with uniform chromatin, fibrosis of the infiltrated zones, a trabecular structure. Spleen positive. Same IPT in BP, and spleen biopsy	Sx (splenectomy)+systemic CS+3 i.t. MTX	Relapse, died, 0.25
[16]	2002	Case 1	69/M	5 years later	Altered mental status, tonic-clonic seizure, weakness, ataxia	Meningeal involvement	CSF: Atypical lymphocytes	Blood, BM, spleen	Oral Dexa. RTx (Cranio-spinal) CTx (2 cycles CVP)	PR, alive
[17]	1994	Case 1	62/M	Simultaneously	Weight loss, fatigue abdominal fullness (splenomegaly)	Meningeal involvement	CSF: typical villous lymphocytes with same surface markers as the lymphocytes in the PB and spleen CD19+, CD20+, HLA-DR+, CD5-, CD10-	Blood, BM, spleen, intraabdominal LN: PB: villous lymphocytes with monoclonal surface Ig with kappa light chain and mu and gamma heavy chains, CD22+, HLA-DR +, CD19+, CD20+, CD3-, CD4-, CD8-; abdominal CT scan: splenomegaly, paraaortic lymphadenopathy, and a tumor around kidneys. BM: 2% villous lymphocytes and a small nodular infiltration; splenectomy: SLVL	Sx (splenectomy)+CS. Relapse: CTx (CHOP14(4 cycles )+ i.t. MTX)	CR, alive, 0.83
Our case	2024	Case 1	50/M	Simultaneously	Orbital apex disorder (cavernous sinus syndrome + Orbital apex syndrome) -spinal LN	Brain CT scan and orbital-cerebral MRI: infiltrating tumor process in the sellar region and cavernous sinuses, orbital pyramids encompassing optic nerves, partially filling the optochiasmatic cistern and extending posteriorly toward the prepontal cistern	-	LN biopsy: MZL B-cells CD20+, CD79a+, Bcl2+, Bcl6+, CD3-, CD5-, CD10-, CD23-, cyclinD1-, SOX11-. Ki-67: 5%. PET-scan: bilateral, symmetrical, retro-ocular orbital hypermetabolism with small pathological hypermetabolic left spinal ganglia	CTx (6 cycles MR-CHOP+2 cycles MR-RCVP)	PR, alive, 0.58

The involvement of the cavernous sinus by high-grade systemic lymphomas is well-documented but has rarely been reported in cases of indolent lymphomas. In PCNSMZL, where dural localization is most commonly described, invasion of the cavernous sinus by contiguity is frequently observed. However, to our knowledge, only a dozen cases with an initial location in the cavernous sinus and no associated dural involvement have been described in the literature, both in PCNSMZL and SCNSMZL [13,33-42]. In the case we report, the process infiltrated the sellar region, including the cavernous sinus, without associated dural involvement or nerve invasion. 

This raises several theories regarding the initial location of the lymphoma: a systemic location with secondary involvement of the cavernous sinus, or conversely, a primary cavernous sinus involvement with the contiguous invasion of the ocular compartments and the intracavernous portion of the carotid artery, followed by systemic dissemination to the optic region, resulting in cavernous sinus syndrome. The latter occurs when one of these contained structures is compromised, usually due to compression, inflammation, or invasion [43,44]. In fact, our patient had a history of chronic smoking and chronic allergic rhinitis, which was self-medicated with corticosteroids, and clinically presented with cavernous sinus syndrome. On the other hand, a chronic inflammatory state can serve as a nidus for MALT lymphoma, or conversely, a consequence of it by recruiting polyclonal lymphocytes from which a monoclonal lymphoma could develop within mucosal sites lacking native lymphoid tissue [15].

Histological evidence of cerebral involvement was obtained through stereotactic biopsy of the tumor mass, surgical removal, or immunophenotyping of CSF in almost half of the cases reported in the literature (Table 1). In our case, immunophenotyping could not be performed due to a lack of resources, and the location of the mass made it difficult to access. Pathological analysis of the spinal adenopathy biopsy, along with the fact that it remained indolent for 12 months, was sufficient to establish the diagnosis of SCNSMZL. Histologically, most SCNSMZLs are of the MALT or nodal MZL subtypes, with only three cases of splenic MZL with secondary cerebral involvement described [24,28,31].

On neuroimaging, most CNSMZLs have a dural location, and the lesions are often indistinguishable from meningiomas, with which they share many features, including prevalence in women, age of onset, and potential multifocality [1,45]. MRI typically reveals single or multiple extra-axial masses with well-defined margins and extensive dural attachment, showing diffuse enhancement upon gadolinium administration and the presence of the "dural tail sign." Most dural lymphomas exhibit a local hyperintensity in the T2/FLAIR signal on MRI (vasogenic edema), with significant enhancement compared to the tumor and adjacent dura, and sometimes erosions of neighboring bones, in contrast to the hyperostosis seen in low-grade meningiomas. Furthermore, other MRI sequences, such as diffusion-weighted imaging (DWI) changes and apparent diffusion coefficient (ADC), can help differentiate between benign meningiomas and dural lymphomas [15,46].

In general, CNS MZBCLs exhibit indolent clinical behavior and a generally favorable prognosis, distinguishing them from aggressive lymphomas that more commonly involve the CNS [22,47,48]. Patients with primary CNSMZL also appear to be more likely to achieve a complete CNS response compared to those with secondary CNSMZL, as well as having better progression-free survival (PFS) and overall survival (OS). This may be due in part to the advanced stage of the disease at diagnosis, poor performance status, general symptoms, and the experience of a PFS event, among other factors [13].

Given the rarity of secondary CNSMZL, conducting randomized controlled trials is challenging. Randomized studies evaluating different treatment modalities have not been published, and therefore, there are no internationally standardized treatments [19,26,27]. Proposed therapeutic strategies or clinical trials logically prioritize aggressive lymphomas that exhibit a poor prognosis in the absence of autologous stem cell transplantation (ASCT) [7,49]. Reported therapeutic options include surgery, radiotherapy [13,19], chemotherapy [13,19,23,25-28], immunotherapy, and combinations of these strategies (chemotherapy and irradiation [17], surgery and radiotherapy [13], and surgery and chemotherapy [13,19,23,29]). Unlike primary CNSMZL, which is radiosensitive and where surgical treatment with or without radiation therapy can be sufficient to control the disease or even achieve complete remission, especially in isolated leptomeningeal lesions, SCNSMZL treatment may require systemic chemotherapy [13,32,37,49]. At least half of the patients received exclusive chemotherapy.

Aggressive chemotherapy approaches have been described, as in the case published by Buseman et al., where an intensive regimen involving drugs with good penetration into the cerebrospinal fluid (CSF), such as MTX, Busulfan, Thiotepa, and Fludarabine, was followed by allogeneic stem cell transplantation from an unrelated donor to achieve a cure for lymphoma [50-52]. The rationale for this approach was based on the atypical clinical characteristics and the potential beneficial effect of the graft-versus-leukemia/lymphoma response, which is essential for eradicating residual hematologic malignancies after allogeneic stem cell transplantation. However, less aggressive approaches with encouraging results have recently been adopted, demonstrating efficacy in the context of a disease with an indolent course despite its atypical cerebral location [13,19,23,25-28]. Different intensity regimens have been used, including chemotherapy protocols (R-CHOP-like regimen, high-dose methotrexate, high-dose cytarabine, fludarabine, ifosfamide, mitoxantrone), immunotherapy (rituximab), and targeted therapy (ibrutinib). Intrathecally administered drugs (methotrexate, dexamethasone, cytarabine) have also been reported, either alone or in combination with intravenous chemotherapy. Other more atypical approaches have been used, either as first-line or relapse treatments, including intrathecal immunotherapy with rituximab, with promising results [23]. Despite the small number of patients, chemotherapy appears to provide a more complete response and better survival rates [13,19,23,26,27].

Because of its ability to cross the blood-brain barrier when doses exceed 560 mg per day, ibrutinib is an interesting option for the treatment of systemic MZL and CNS involvement in other NHL cases. In the series by Furqan et al., three of the four patients treated for SCNSMZL with an ibrutinib-based regimen (Ibrutinib + Rituximab; Ibrutinib + Rituximab + Temozolomide; Ibrutinib + Bendamustine + Obinutuzumab) had PFS and OS in the CNS after ibrutinib treatment, ranging from one to three years [16]. CAR T cells and hematopoietic stem cell allografts, except for the one case published by Buseman et al., are still used in rare cases of transformation into aggressive MZL [53,54].

Our case study has certain limitations. The use of corticosteroids prior to the first consultation prevented us from characterizing all the organs initially affected. Additionally, we did not perform a stereotactic biopsy to obtain a sample due to the difficult-to-access location, the indolent course of the disease, and the rapid response to corticosteroids, which supported our initial diagnosis. Bone marrow examination was not initially performed, and the CT scan was replaced by a PET scan. The partial response to chemotherapy and the stable nature of the disease will lead to discussions regarding a possible combination therapy, including ibrutinib with or without rituximab, in the event of disease progression.

## Conclusions

In summary, secondary CNS involvement by MZBCL is rare, and localization in the cavernous sinus is exceptional. This type of lymphoma typically has an indolent clinical course and a generally favorable prognosis, highlighting the importance of differentiating it from meningiomas in cases of meningeal localization, and from aggressive lymphomas, which more commonly involve the CNS. The use of less aggressive therapeutic approaches, particularly in the era of immunotherapy and targeted therapies, will enable prolonged remission with reduced toxicity, especially in younger patients.
